# Denis pattern–dependent biomechanical behavior of posterior trans-iliac plate fixation compared with bilateral triangular osteosynthesis in unilateral sacral fractures: a finite element study

**DOI:** 10.1007/s00068-026-03238-w

**Published:** 2026-06-12

**Authors:** Jie Yang, Kai O. Böker, Chun Li, Arndt F. Schilling, Zully Ritter, Mehool Acharya, Wolfgang Lehmann

**Affiliations:** 1 Department of Trauma Surgery, Orthopaedics and Plastic Surgery, University Medical Center Göttingen, Robert Koch Straße 40, 37075 Göttingen, Germany; 2 Central Emergency Department, University Medical Center Göttingen, Robert-Koch Straße 40, 37075 Göttingen, Germany; 3 Campus Institute Data Science, University of Göttingen, Göttingen, Germany; 4 Department of Trauma and Orthopaedic Surgery, Nottingham University Hospitals NHS Trust, Nottingham, UK

**Keywords:** Sacral fracture, Denis classification, posterior pelvic ring, trans-iliac plate osteosynthesis, triangular osteosynthesis, finite element analysis

## Abstract

**Objective:**

Posterior trans-iliac plate osteosynthesis (TPO) is commonly used for the stabilization of unilateral vertically unstable sacral fractures. However, its biomechanical performance across different Denis fracture patterns remains unclear. This study aimed to investigate the fracture pattern–dependent biomechanical behavior of posterior trans-iliac plate osteosynthesis (TPO) in unilateral vertically unstable sacral fractures, using bilateral triangular osteosynthesis (BTO) as a high-stability reference construct.

**Methods:**

A validated three-dimensional finite element model of the lumbopelvic complex was developed to simulate unilateral vertically unstable sacral fractures corresponding to Denis type I, II, and III patterns. Two posterior fixation strategies, TPO and BTO, were constructed for each fracture type. Seven physiological loading conditions were applied, including standing, flexion, extension, axial rotation, and lateral bending. Von Mises stress distribution and fracture displacement were evaluated. Fracture micromotion was quantified by calculating relative displacement changes between paired points along the fracture gap.

**Results:**

When interpreted relative to the high-stability BTO reference construct, TPO showed fracture pattern–dependent variation in fracture displacement. The difference was most pronounced in Denis type I fractures and progressively decreased in Denis type II and III patterns.

**Conclusion:**

The biomechanical performance of posterior trans-iliac plate fixation is influenced by the Denis fracture pattern. Fracture micromotion associated with the non-reinforced TPO construct evaluated in this study decreased as the fracture line approached the sacral midline, indicating improved mechanical compatibility in more medially located fracture patterns. These findings provide biomechanical reference data for understanding fracture pattern–dependent fixation compatibility of TPO in unilateral sacral fractures. However, it remains unclear how much fixation strength is actually required to achieve sufficient construct stability for fracture healing and how postoperative management strategies, such as weight-bearing status, may influence this requirement.

## Introduction

Sacral fractures involving the posterior pelvic ring remain a major clinical challenge due to their inherent instability and substantial impact on patient mobility and functional recovery [1]. Among vertically unstable pelvic ring injuries classified as AO/OTA type C, unilateral patterns are particularly common [2]. Previous clinical series have reported that fractures with unilateral vertical instability, especially type C1 and C2 injuries, account for approximately three quarters of all type C pelvic fractures. In these cases, the predominant injury pattern involves unilateral disruption of the sacrum [3–5].

To describe fracture location and predict neurological injury, the Denis classification system subdivides sacral fractures into zones I–III based on their relationship to the sacral foramina and central canal [6]. While this classification has been widely adopted in clinical practice for neurological risk assessment, its biomechanical implications have received considerably less attention. In particular, it remains unclear whether different posterior fixation strategies exhibit consistent biomechanical performance across various Denis fracture patterns.

In current clinical practice, various posterior pelvic ring fixation constructs are utilized for the treatment of unilateral vertically unstable sacral fractures [7–10]. Posterior trans-iliac plate osteosynthesis (TPO) represents a direct posterior ring stabilization strategy, in which a plate is contoured and positioned transversely across the posterior ilium to function as a tension-band construct [11]. With advances in surgical techniques, this method has been progressively adapted into percutaneous and minimally invasive approaches, allowing posterior ring stabilization with reduced surgical exposure and soft-tissue disruption and complications [12].

Triangular osteosynthesis was originally introduced as a combined fixation concept integrating lumbo-pelvic instrumentation with sacroiliac screw fixation, aiming to enhance stability in vertically unstable sacral fractures [13]. Mechanically linking the lumbar spine to the pelvis and simultaneously stabilizing the sacral fracture, allows this construct to provide a distinct load-sharing pathway across the lumbopelvic junction [14]. When applied bilaterally, triangular osteosynthesis further reinforces posterior pelvic ring stability by symmetrically distributing vertical and rotational loads. As a result, bilateral triangular osteosynthesis (BTO) is widely regarded as one of the most mechanically reliable fixation strategies for unstable sacral fractures involving the posterior pelvic ring.

Therefore, the present study aimed to investigate the biomechanical behavior of TPO across different Denis fracture patterns in unilateral vertically unstable sacral fractures, using BTO, a widely recognized high-stability fixation construct, as a mechanical reference framework.

## Materials and methods

### Geometric reconstruction

This study was approved by the Medical Ethics Committee of the University Medical Center Göttingen and was conducted in accordance with the Declaration of Helsinki and its later amendments. Computed tomography (CT) data were obtained from a healthy adult male volunteer with no history of spinal or pelvic pathology. All images were stored in Digital Imaging and Communications in Medicine (DICOM) format.

**Fig. 1:**
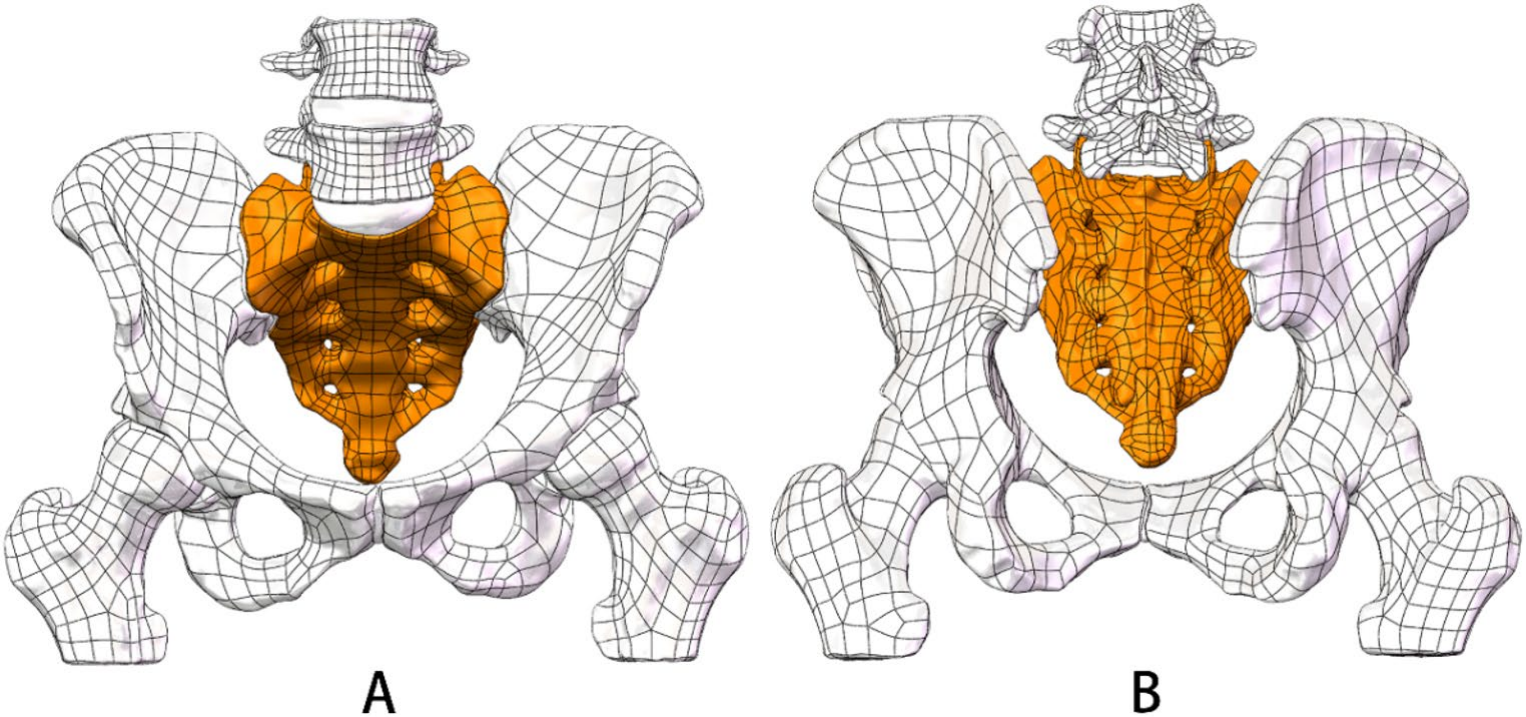
Three-dimensional model representation of the pelvis used in the present study. The sacrum (highlighted in orange) represents the primary region of interest. Anterior (**A**) and posterior (**B**) views are presented to demonstrate the overall geometry and anatomical alignment of the pelvic ring

Image segmentation of the lumbar spine, pelvis, and proximal femora was performed using 3D Slicer (version 5.11.0). The reconstructed bony structures were subsequently processed in Geomagic Wrap (2021) to remove artifacts and ensure smooth surface continuity. The refined geometries were then imported into SolidWorks 2025, where a complete three-dimensional lumbopelvic–femoral model in the intact condition was developed (Fig. 1).

### Fracture modeling

**Fig. 2:**
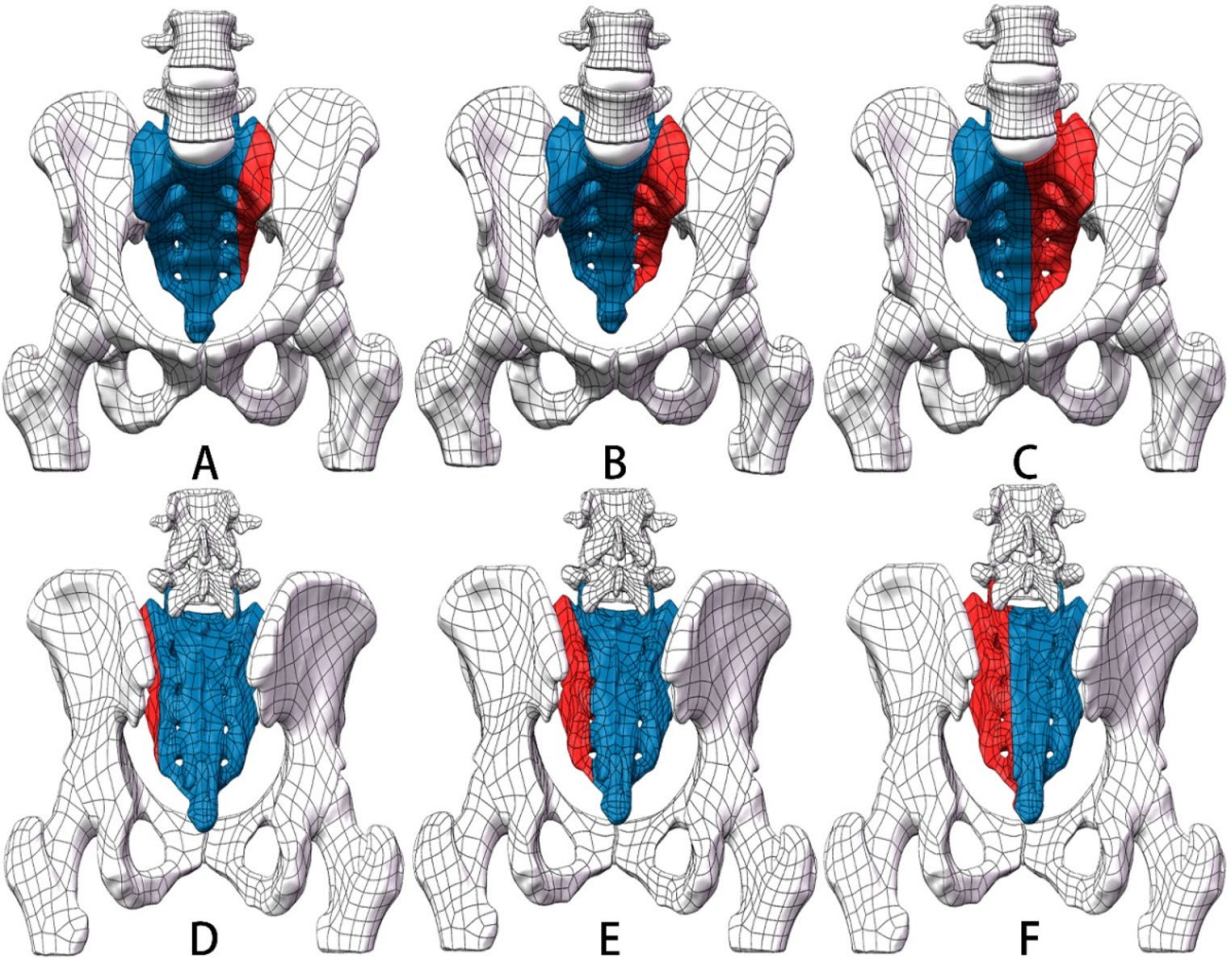
Schematic illustration of unilateral sacral fracture models according to the Denis classification. Fracture lines were created on the left side of the sacrum to represent Denis type I (**A**, **D**), type II (**B**, **E**), and type III (**C**, **F**) fractures, corresponding to different anatomical relationships with the sacral foramina and central canal. Anterior (upper row) and posterior (lower row) views are shown

To represent vertically unstable unilateral sacral injuries, three fracture models were constructed according to the Denis classification system [6]. Longitudinal fracture lines were geometrically created on the left side of the sacrum in SolidWorks, and the sacral fragments were separated into independent bodies corresponding to Denis type I, II, and III patterns. The models were then imported into ANSYS for finite element analysis. Surface-to-surface frictional contact was defined between the opposing fracture surfaces, allowing relative motion while preventing penetration. A friction coefficient of 0.4 was assigned to simulate bone-to-bone interaction. No reduction of elastic modulus or artificial softening was applied at the fracture interface. (Fig. 2A–F).

All fracture patterns were designed to represent posterior pelvic ring instability with vertical shear characteristics. In all models, the anterior pelvic ring remained intact in order to minimize the influence of anterior ring disruption on posterior fixation behavior and to allow a more controlled evaluation of posterior construct biomechanics across different Denis fracture patterns. This simplified configuration was intended to isolate the biomechanical influence of posterior fixation constructs under controlled conditions.

### Internal fixation constructs

All internal fixation constructs were created in SolidWorks 2025 based on standard surgical dimensions and commonly applied clinical techniques.

#### Posterior trans-iliac plate osteosynthesis

A posterior trans-iliac plate osteosynthesis (TPO) construct was established based on previously reported techniques of posterior pelvic ring tension-band plating. A straight posterior trans-iliac plate construct was modeled to simulate tension-band fixation. The plate had a width of 14 mm and a thickness of 6 mm, and its length was determined to allow secure bilateral iliac fixation. It was positioned between the bilateral posterior superior iliac spines and aligned along the superior margin of the sacral foramina.

**Fig. 3:**
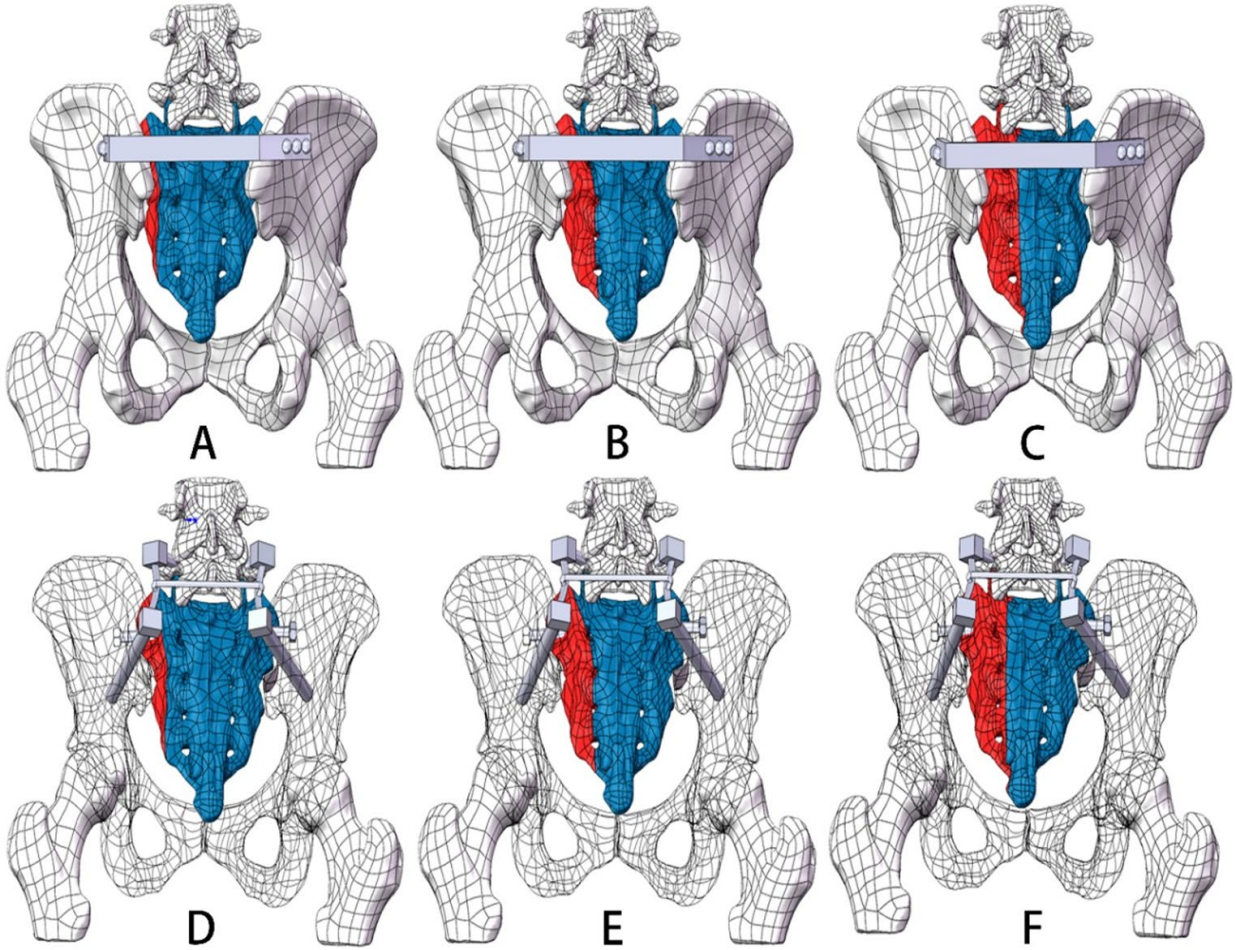
Three-dimensional models of posterior pelvic ring fixation across unilateral sacral fractures of different Denis types. For both fixation strategies, the fracture pattern is arranged from left to right as Denis type I, type II, and type III. TPO (**A**–**C**); BTO (**D**–**F**)

Three 4.5 mm diameter screws were inserted on each side to secure the plate to the bilateral ilia. The screws crossed the sacroiliac joints at the sacral cortical level without penetrating the sacral fracture surfaces. This configuration was designed to stabilize the posterior pelvic ring through a trans-iliac tension-band mechanism (Fig. 3A–C).

This construct was modeled as a standardized posterior trans-iliac tension-band configuration. It does not represent all possible TPO variations, such as reinforced constructs with additional iliosacral screws, longer screw trajectories, or double-plate fixation.

#### Bilateral triangular osteosynthesis

The bilateral triangular osteosynthesis (BTO) model consisted of bilateral lumbo-pelvic fixation combined with bilateral sacroiliac screw fixation. Pedicle screws were inserted into the L5 vertebra, and iliac screws were placed bilaterally through the posterior superior iliac spines into the ilium. In addition, bilateral S1 sacroiliac screws (80 mm length, 7.3 mm diameter) were positioned across the sacroiliac joints and passed through the fracture line to provide direct stabilization of the posterior pelvic ring and improve fracture-site stability.

This construct provided stabilization of the posterior pelvic ring through both sacroiliac fixation and the lumbopelvic fixation pathway (Fig. 3D–F). All implants were assembled in SolidWorks 2025 prior to finite element analysis. In the present study, BTO was modeled as a high-stability reference construct to contextualize the biomechanical behavior of TPO across different Denis fracture patterns. It was not intended to represent a clinically equivalent alternative for all unilateral sacral fractures.

### Mesh generation and material properties

The assembled bone–implant models were imported into ANSYS 2025R2 for finite element analysis. Three-dimensional quadratic tetrahedral elements (ANSYS element type: SOLID187; 10-node elements with quadratic interpolation functions) were used for all bone and osteosynthesis construct components. Mesh refinement was performed to ensure numerical stability. A mesh convergence analysis was conducted to verify the numerical reliability of the finite element model. When the mesh density was refined beyond the medium level, the displacement variation was less than 5%, indicating that mesh convergence had been achieved. Based on this analysis, the medium mesh density was adopted for all subsequent simulations. The number of finite elements, nodes, and contact interfaces in the intact pelvis model are summarized in Table 1.

**Table 1:** Number of elements and nodes in the intact and fixation FE models

Model	elements	nodes
Intact model	321,377	498,423
Denis I fracture	328,148	502,583
Denis II fracture	327,988	502,148
Denis III fracture	329,525	502,363
TPO Denis I	398,729	557,583
TPO Denis II	398,527	553,527
TPO Denis III	401,427	561,239
BTO Denis I	404,283	563,643
BTO Denis II	417,849	586,229
BTO Denis III	402,643	563,534

Material properties for cortical bone, cancellous bone, intervertebral discs, and metallic implants were assigned based on values reported in previous biomechanical studies [15–18]. All materials were assumed to be homogeneous, isotropic, and linearly elastic. Detailed parameters are summarized in Table 2.

**Table 2:** Material properties of finite element method (FEM) models

Material	Elastic modulus, MPa	Poisson ratio
Cortical bone (Lumbar)	12,000	0.3
Cancellous bone (Lumbar)	345	0.2
Cortical bone (Ilium)	17,000	0.3
Cancellous bone (Ilium)	132	0.2
Cortical bone (Sacrum)	6140	0.3
Cancellous bone (Sacrum)	1400	0.3
Disc (Annulus)	8.4	0.45
Disc (Nucleus)	Mooney–Rivlin c1 = 0.12, c2 = 0.03	
Articular cartilage	100	0.3
Implants (Ti)	110,000	0.3

### Contact definitions and ligament modeling

The interfaces between screws and surrounding bone were defined using shared nodes, representing rigid fixation without relative motion.

Plate–bone interfaces were modeled using surface-to-surface contact elements, allowing limited sliding between the metallic plate and adjacent bone. A friction coefficient of 0.45 was applied.

Pelvic ligaments were represented using linear elastic spring elements. Each ligament was modeled as a tension-only linear spring with stiffness values obtained from previously published biomechanical studies [15–18]. The springs were assigned unidirectional axial stiffness without nonlinear behavior. Detailed parameters are summarized in Table 3.

**Table 3:** Model properties of ligament

Ligaments	K, *N*/mm	Number of springs
Anterior sacroiliac	1500	30
Interspinous	3000	15
Long posterior sacroiliac	10,000	8
Short posterior sacroiliac	7500	30
Sacrospinous	8000	9
Superior pubic ligament	250	12
Arcuate pubic ligament	250	12

To reduce computational complexity, screw threads were omitted, and all screws were modeled as smooth cylinders with diameters corresponding to their outer thread diameters.

### Boundary and loading conditions

**Fig. 4:**
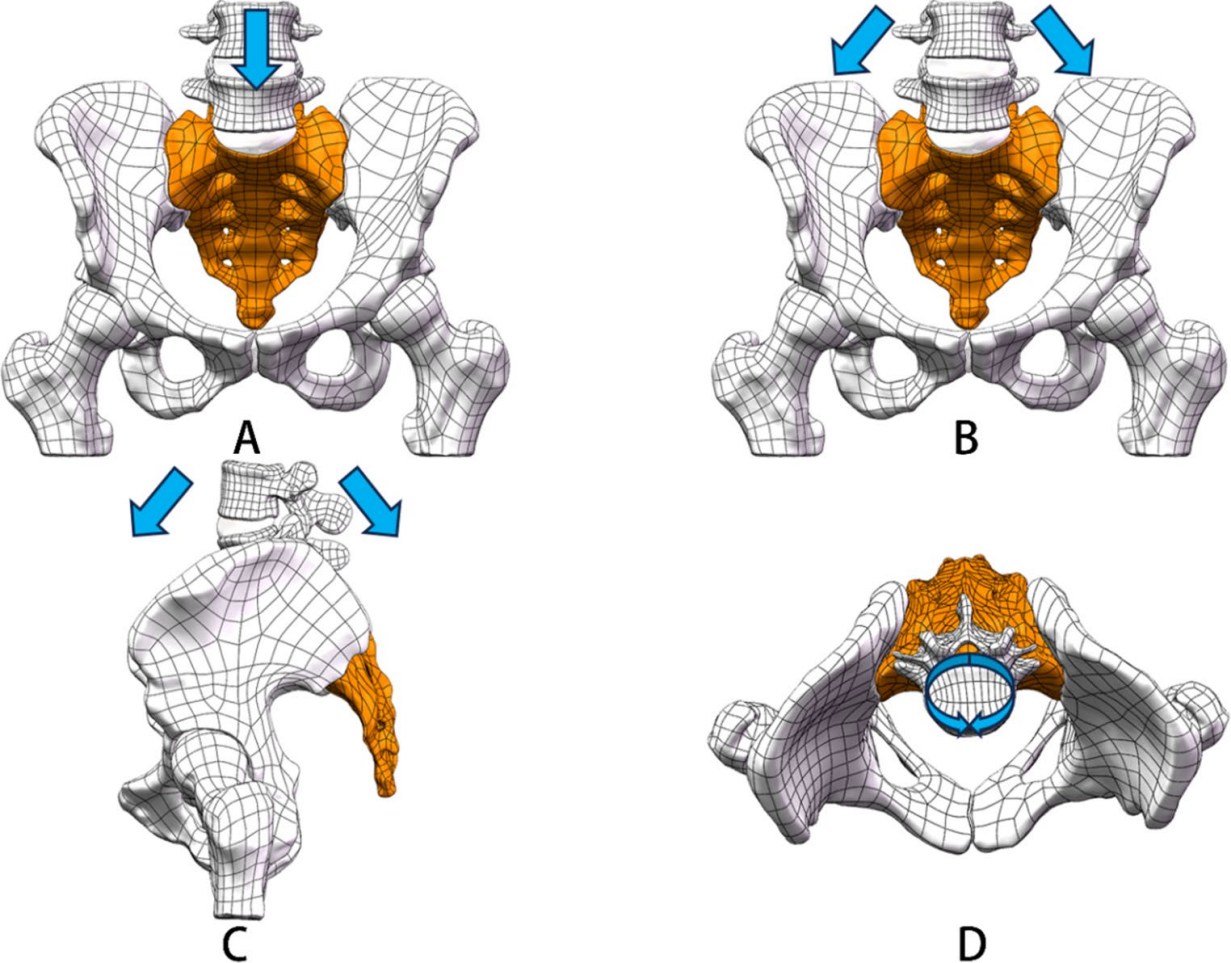
Different movements of the model (**A**) standing (pure compression); (**B**) lateral flexion; (**C**) flexion and extension and (**D**) rotation

Symmetrical fixed boundary conditions were applied at the distal ends of both femora to simulate bilateral lower-limb support and to ensure structural continuity of the lumbopelvic–femoral complex during load transmission. A vertical compressive preload of 600 N was applied to the superior surface of the L4 vertebral body to simulate the upper-body weight transmitted through the lumbopelvic complex during upright standing (Fig. 4A). The magnitude of 600 N has been widely adopted in validated spinal and lumbopelvic finite element studies to represent physiological axial loading under neutral standing conditions [19–21].

Under the same loading framework, a pure moment of 10 N·m was superimposed on the 600 N axial preload to simulate physiological motions of the lumbopelvic complex. This combined loading protocol, consisting of a constant compressive preload and an applied moment, has been widely used in validated spinal and lumbopelvic finite element studies to represent functional movements under weight-bearing conditions [22–24]. The applied moments generated different postural configurations, including lateral bending (Fig. 4B), flexion and extension (Fig. 4C), and axial rotation (Fig. 4D).

### Outcome measurements

Von Mises stress distribution within the implants and posterior pelvic ring was analyzed under all loading conditions. For quantitative comparison, mean von Mises stress values of the fixation constructs were extracted and compared across different fracture patterns and loading scenarios. Fracture displacement was quantified by evaluating changes in the relative distance between paired reference points located on both sides of the sacral fracture.

**Fig. 5:**
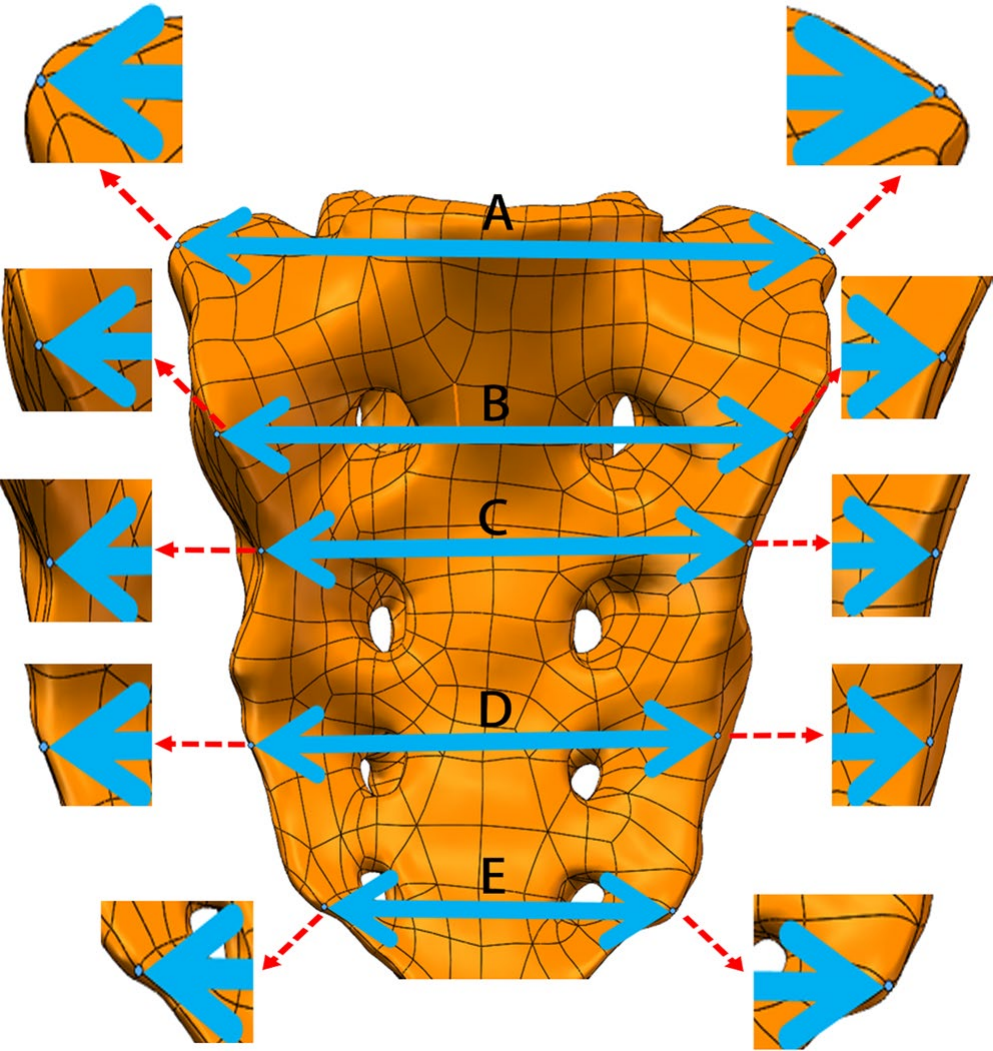
Schematic illustration of fracture gap micromotion measurement. Five transverse measurement levels in the sacrum (**A**–**E**) from cranial to caudal were defined to calculate the relative displacement between the bilateral fracture surfaces at each level

Five pairs of corresponding anatomical reference points (**A–E**, Fig. 5) were predefined bilaterally along the fracture line from the superior to the inferior sacral region. These reference points were defined on the original geometric model prior to mesh generation and were preserved during transfer to ANSYS.

For each loading condition, the Euclidean distance between each pair of reference points was calculated in the initial unloaded configuration and after load application. Displacement values were extracted at the mesh nodes corresponding to these predefined geometric locations to ensure consistent anatomical positioning across simulations. The difference between the two distances was defined as the fracture displacement at that level.

As the present study represents a deterministic finite element analysis, each loading condition generated a single computational solution under predefined parameters. Therefore, the results were interpreted descriptively, and no statistical inference or hypothesis testing was performed.

### Model validation

To ensure the reliability of the FE model used in the present study, a two-step validation strategy was applied. First, the intact lumbopelvic model adopted in this study was previously established and validated in our earlier investigation, in which its load–displacement behavior was compared with cadaveric experimental data reported by Takayama et al. [25, 26]. Detailed validation procedures and numerical comparisons have been described in that study. The previously validated model framework was subsequently adopted for the present investigation.

Second, to further evaluate the stress distribution characteristics of the present model, an additional stress-based validation was performed following the methodology reported by Dalstra et al. [27], which provides experimentally measured cortical stress data at predefined strain-gauge locations under a vertical compressive loading. A pure vertical compression load of 600 N was applied, and von Mises stress values were extracted from eight cortical surface regions corresponding to the strain-gauge positions described in the original experimental setup.

In the present model, these regions were identified using reproducible anatomical landmarks consistent with the locations reported by Dalstra et al., including the superior acetabular rim, the medial cortex beneath the acetabular dome, the pubic bone, and the region of the greater sciatic notch. Stress values were obtained from cortical surface elements within these anatomically corresponding regions. The resulting stress values were subsequently compared with the experimental measurements and the two reference FE models (realistic heterogeneous and homogeneous pelvis models) reported by Dalstra et al.

**Fig. 6:**
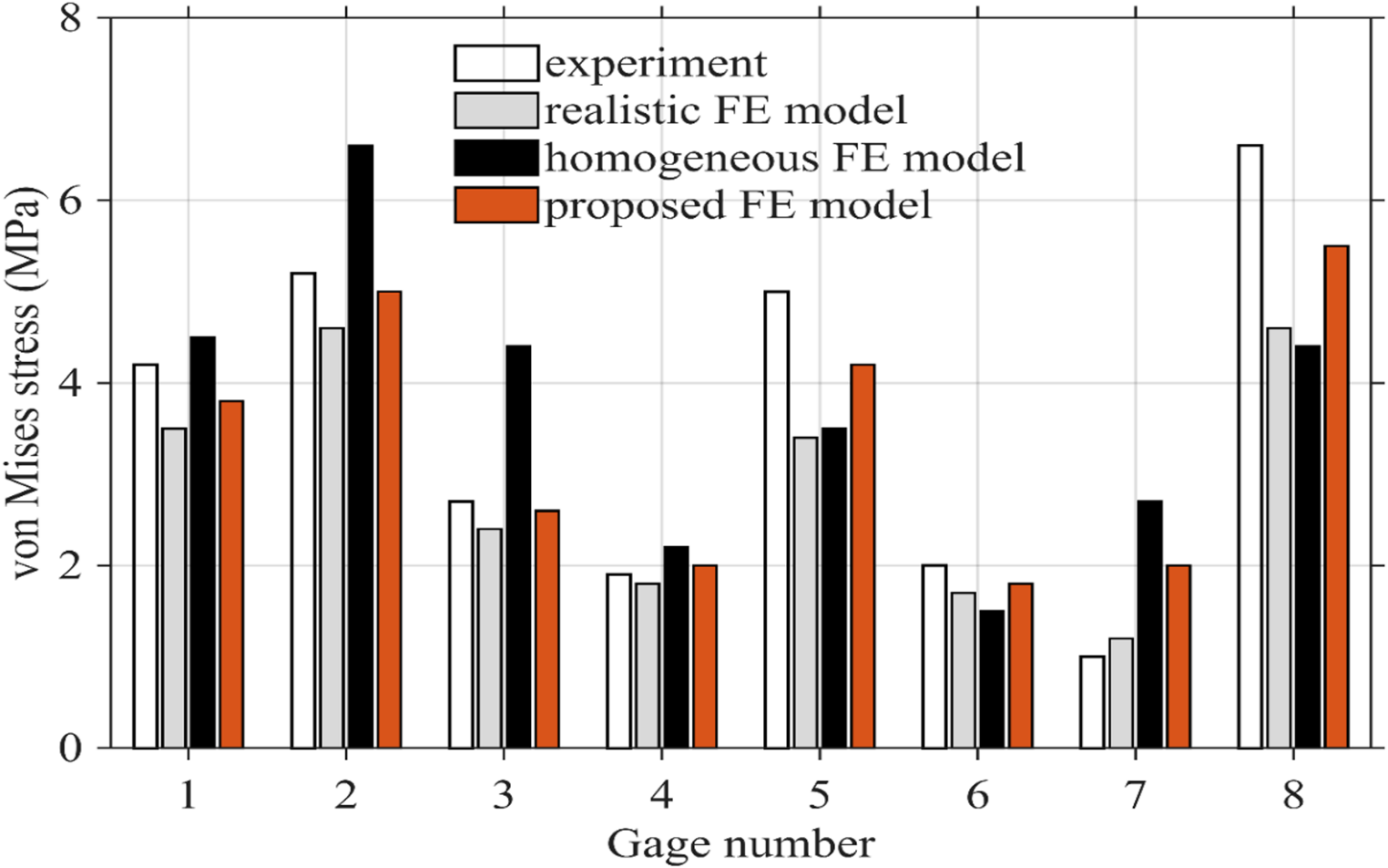
Model validation under a compressive load of 600 N. Von Mises stress values measured at eight-gauge locations are compared among experimental data and FE models reported in the literature, including a realistic heterogeneous model and a homogeneous model. The proposed finite element model developed in the present study is shown in red. Stress values were extracted from cortical surface regions corresponding to the anatomical strain-gauge locations reported by Dalstra et al. [27]

Comparable stress magnitudes and distribution trends across the eight measurement locations demonstrated reasonable agreement between the present model and the previously published experimental and finite element data, supporting the suitability of the model for subsequent biomechanical comparisons (Fig. 6).

## Results

### Stress distribution

**Fig. 7:**
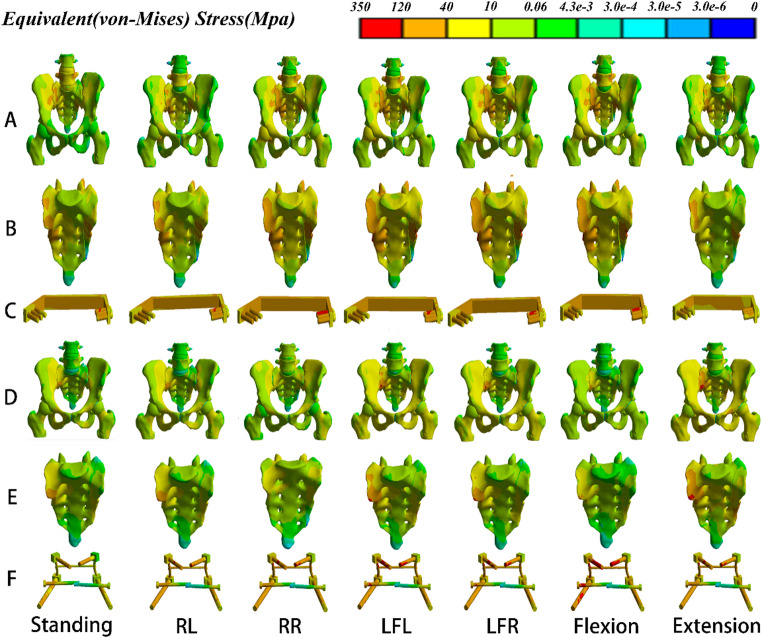
Von Mises stress distribution of the pelvic model with a Denis type I unilateral sacral fracture under different loading conditions. For this fracture type, stress contours are presented in six rows: the entire pelvis (row **A**), the sacrum (row **B**), and the internal fixation construct (row **C**) for the TPO configuration, followed by the corresponding views for the BTO configuration (rows **D**–**F**). From left to right, the loading conditions include standing, rotation left (RL), rotation right (RR), lateral flexion Left (LFL), and lateral flexion right (LFR), flexion, extension

**Fig. 8:**
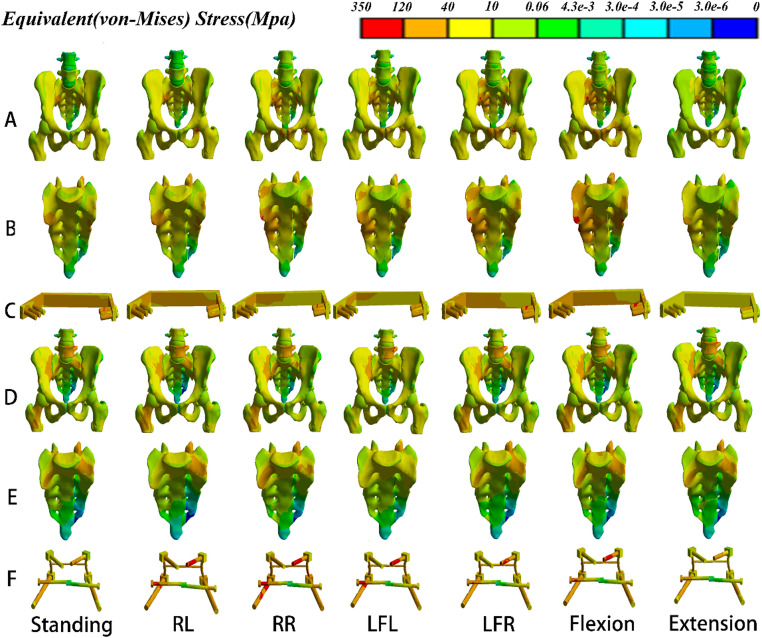
Von Mises stress distribution of the pelvic model with a Denis type II unilateral sacral fracture under different loading conditions. For this fracture type, stress contours are presented in six rows: the entire pelvis (row **A**), the sacrum (row **B**), and the internal fixation construct (row **C**) for the TPO configuration, followed by the corresponding views for the BTO configuration (rows **D**–**F**). From left to right, the loading conditions include standing, RL, RR, LFL, LFR, flexion, extension

**Fig. 9:**
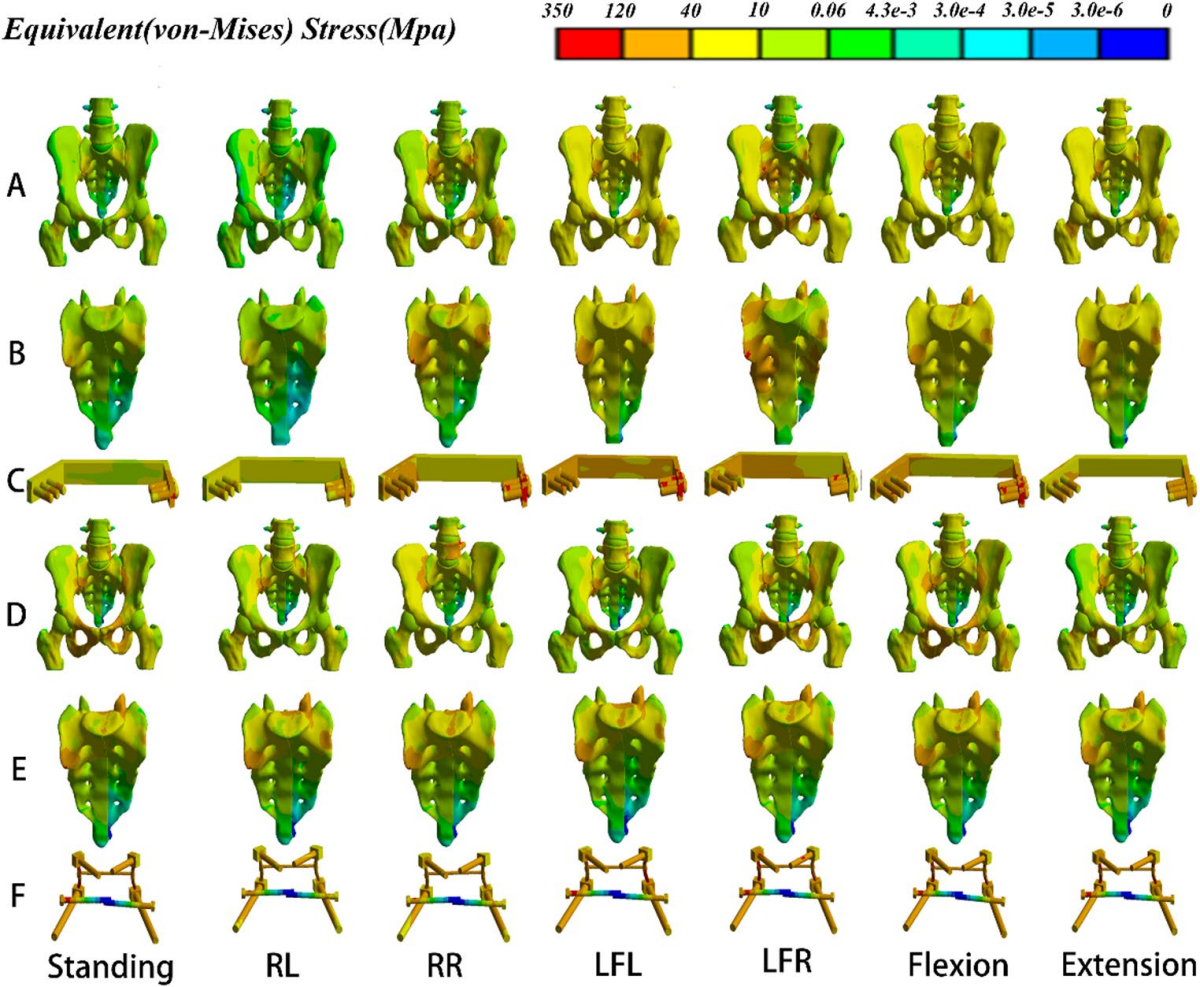
Von Mises stress distribution of the pelvic model with a Denis type III unilateral sacral fracture under different loading conditions. For this fracture type, stress contours are presented in six rows: the entire pelvis (row **A**), the sacrum (row **B**), and the internal fixation construct (row **C**) for the TPO configuration, followed by the corresponding views for the BTO configuration (rows **D**–**F**). From left to right, the loading conditions include standing, RL, RR, LFL, LFR, flexion, extension

The von Mises stress distributions of the two fixation constructs under all loading conditions are shown in Figs. 7, 8 and 9, corresponding to Denis type I (Fig. 7), type II (Fig. 8), and type III (Fig. 9) fractures, respectively. Each figure includes views of the entire pelvis, the sacrum, and the internal fixation devices.

**Fig. 10:**
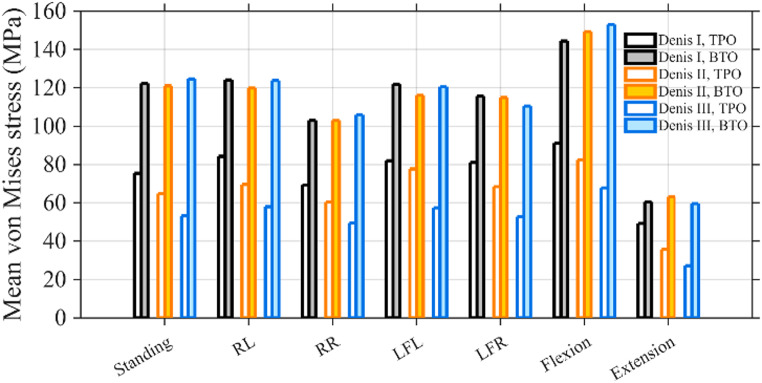
Comparison of mean von Mises stress in the fixation constructs under different loading conditions. Results are presented for unilateral sacral fractures of Denis type I, type II, and type III treated with TPO or BTO. Loading conditions include standing, RR, RL, LF_L, LF_R, flexion, and extension

For both fixation strategies, flexion consistently produced the highest von Mises stress values, whereas extension resulted in the lowest stress levels. This trend was observed in Denis I, Denis II, and Denis III fracture models. Quantitative analysis of the mean von Mises stress of the internal fixation is summarized in Fig. 10.

In Denis I fractures, the mean implant von Mises stress of the TPO ranged from approximately 70–90 MPa under standing and rotational postures, increasing to approximately 90 MPa under flexion. In contrast, the bilateral triangular osteosynthesis (BTO) construct exhibited higher stress levels, with mean values ranging from approximately 120 MPa to 145 MPa, reaching the maximum under flexion.

In Denis II fractures, the TPO demonstrated mean von Mises stress values of approximately 60–85 MPa, whereas the BTO construct showed values ranging from 115 to 150 MPa, with the highest stress again observed under flexion.

In Denis III fractures, the TPO group mean values are generally below 70 MPa in most postures. In contrast, the BTO construct maintained relatively stable stress levels across different fracture types, with mean von Mises stress values ranging from 110 to 155 MPa.

### Fracture displacement

**Fig. 11:**
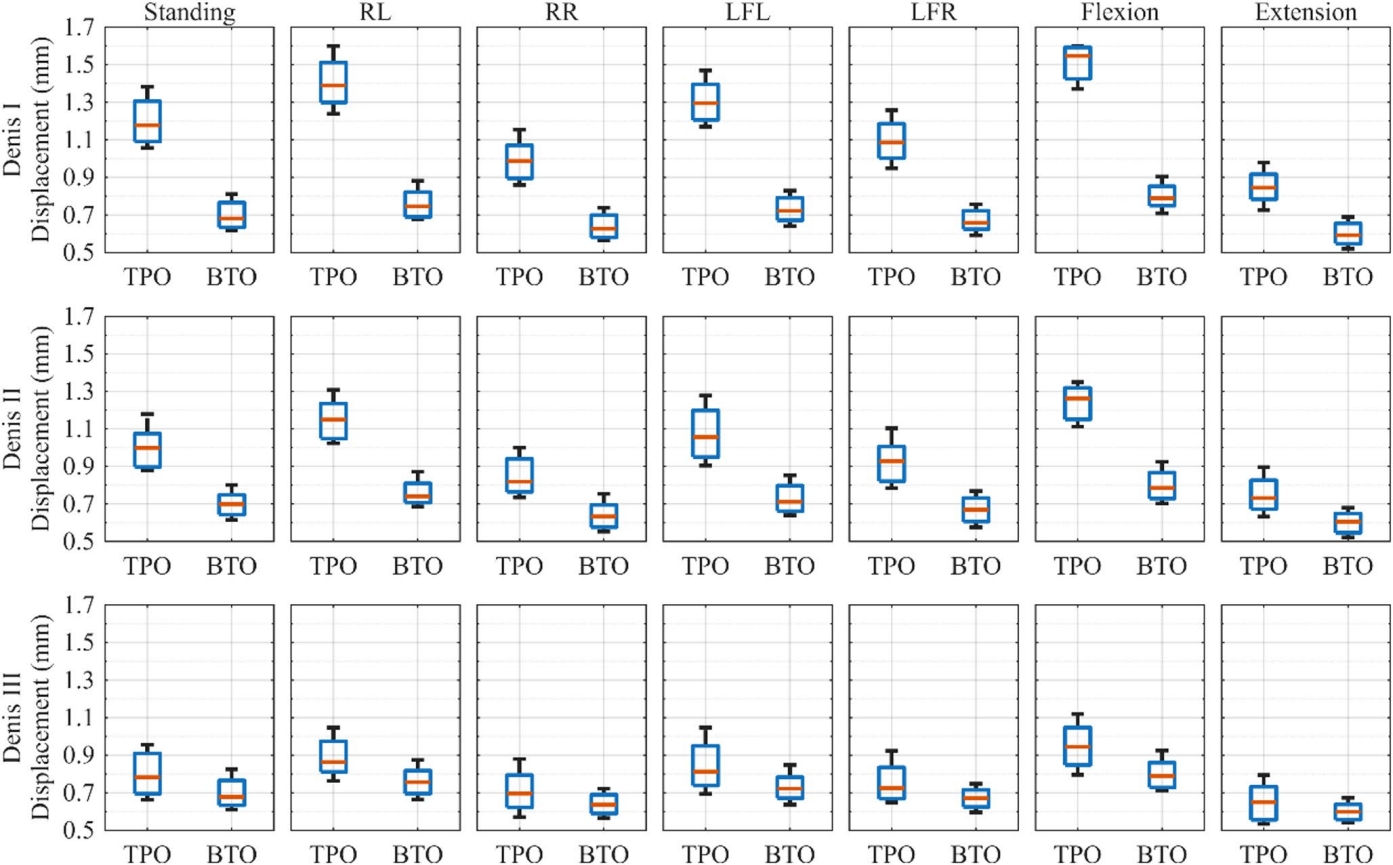
Displacement distribution of the posterior pelvic ring under different loading conditions. Box plots illustrate the distribution of fracture displacement values across the five predefined fracture-gap measurement levels (**A**–**E**) for unilateral sacral fractures of Denis type I, type II, and type III treated with TPO or BTO. For each Denis type, loading conditions are arranged from left to right as standing, left rotation (LR), right rotation (RR), left lateral flexion (LFL), right lateral flexion (LFR), flexion, and extension

Fracture displacement was evaluated under all loading conditions for both fixation constructs. The displacement distribution across five paired points along the sacrum is illustrated using box plots in Fig. 11, and the corresponding mean ± standard deviation values are summarized in Table 4.

In Denis type I fractures, the TPO group demonstrated greater fracture displacement than the BTO group under all loading conditions. The highest displacement values were observed under flexion and left rotation in the TPO group, while the BTO group showed comparatively lower displacement values across the same loading conditions. The overall displacement magnitude was greatest in Denis type I fractures among the three Denis patterns.

**Table 4:** Mean fracture displacement values under different loading conditions

Denis type	Loading condition	TPO, mean ± SD (mm)	BTO, mean ± SD (mm)
Denis I	Standing	1.21 ± 0.22	0.70 ± 0.11
	RL	1.47 ± 0.25	0.76 ± 0.08
	RR	0.95 ± 0.18	0.64 ± 0.07
	LFL	1.28 ± 0.22	0.73 ± 0.08
	LFR	1.15 ± 0.22	0.67 ± 0.06
	Flexion	1.59 ± 0.29	0.80 ± 0.07
	Extension	0.84 ± 0.14	0.60 ± 0.07
Denis II	Standing	1.00 ± 0.10	0.70 ± 0.07
	RL	1.15 ± 0.11	0.79 ± 0.07
	RR	0.83 ± 0.09	0.64 ± 0.06
	LFL	1.05 ± 0.11	0.74 ± 0.07
	LFR	0.93 ± 0.10	0.68 ± 0.06
	Flexion	1.23 ± 0.10	0.82 ± 0.07
	Extension	0.76 ± 0.09	0.61 ± 0.05
Denis III	Standing	0.80 ± 0.10	0.70 ± 0.07
	RL	0.89 ± 0.10	0.77 ± 0.06
	RR	0.72 ± 0.10	0.64 ± 0.05
	LFL	0.84 ± 0.11	0.73 ± 0.07
	LFR	0.78 ± 0.09	0.67 ± 0.05
	Flexion	0.95 ± 0.10	0.80 ± 0.06
	Extension	0.64 ± 0.08	0.60 ± 0.04

In Denis type II fractures, fracture displacement remained greater in the TPO group than in the BTO group across all loading conditions. Compared with Denis type I fractures, the overall displacement values in the TPO group were lower, whereas the BTO group showed relatively stable displacement values across the simulated loading scenarios.

In Denis type III fractures, both fixation constructs demonstrated smaller fracture displacement values than those observed in Denis type I fractures. The displacement values of the TPO group approached those of the BTO group more closely across the loading conditions, indicating a reduced displacement discrepancy in this fracture pattern.

### Difference in fracture displacement between fixation constructs

**Fig. 12:**
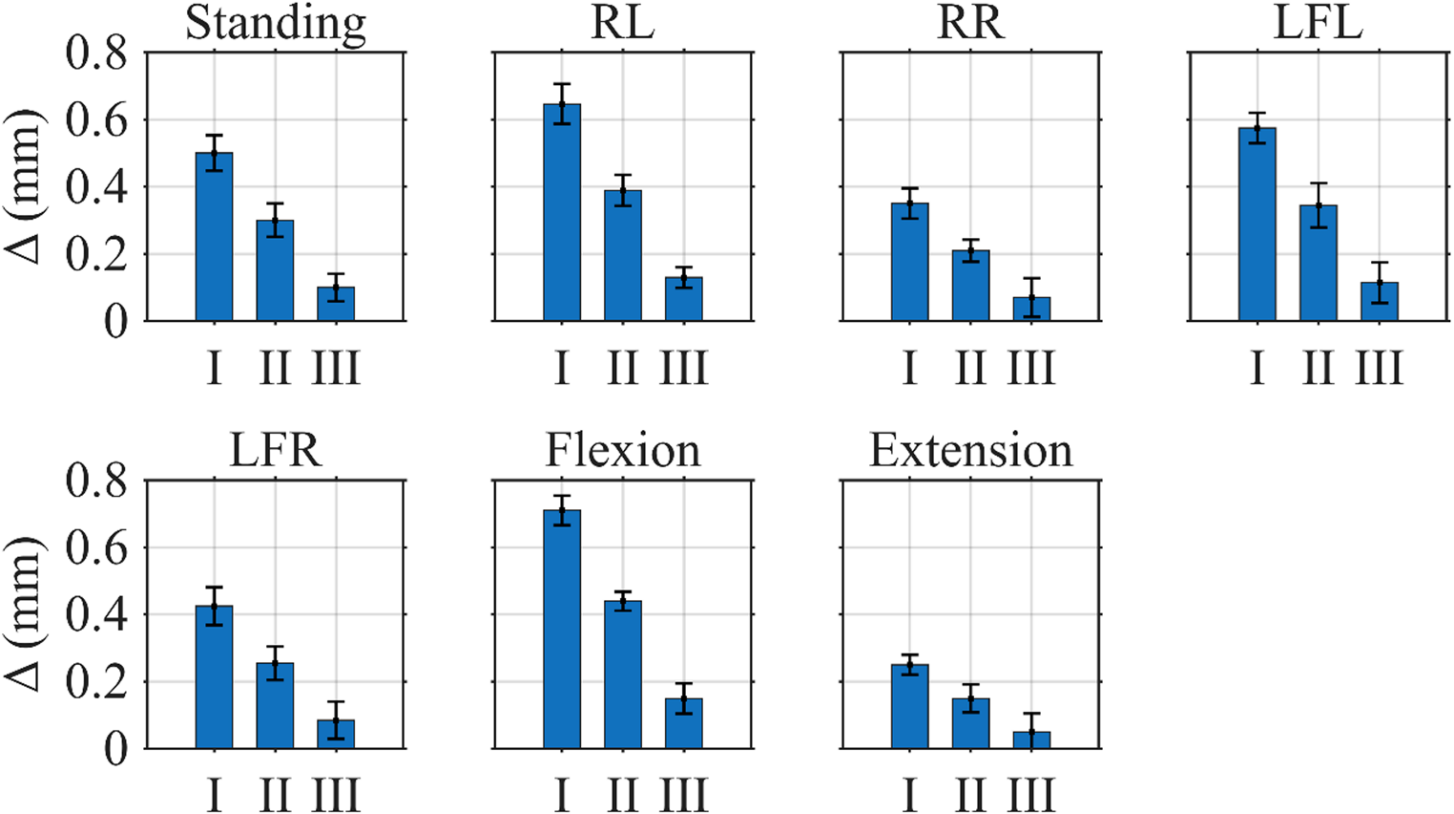
Quantitative comparison of fracture displacement between TPO and BTO under identical loading conditions. The displacement difference (Δ displacement = plate − BTO) is shown for Denis type I, type II, and type III unilateral sacral fractures across standing, rotation, lateral flexion, flexion, and extension loading scenarios. Error bars indicate the standard deviation

The differences in fracture displacement between TPO and BTO under identical loading conditions were further quantified. The displacement difference (Δ displacement = plate − BTO) across Denis fracture types is presented in Fig. 12.

Across all postures, the magnitude of displacement difference decreased progressively from Denis I to Denis III fractures. The largest displacement differences were observed in flexion and rotational postures in Denis I fractures, whereas minimal differences were observed in Denis III fractures, particularly under extension loading.

## Discussion

The present study was designed to investigate whether the biomechanical behavior of TPO varies across Denis patterns in unilateral vertically unstable sacral fractures. BTO was used as a high-stability reference construct, not as a clinically equivalent comparator, to provide mechanical context for interpreting the relative behavior of TPO. Therefore, the primary focus of this study was not to determine which construct is clinically superior, but to clarify how fracture morphology influences the mechanical compatibility of TPO.

Previous finite element work has suggested that the mechanical behavior of TPO may vary across Denis fracture patterns. In a parallel finite element and retrospective clinical analysis, Chen et al. compared percutaneous posterior-ring tension-band plate fixation with percutaneous iliosacral screw fixation for Denis I–III vertical sacral fractures and reported the plate performed better in the Denis type III situation [28]. However, that study used iliosacral screws as the comparator, relied largely on global pelvic-ring displacement metrics (e.g., UR_max) and a limited set of loading conditions, which may not fully capture fracture-gap specific micromotion under a broader spectrum of physiological postures. In contrast, the present study benchmarked posterior trans-iliac tension-band plate fixation against bilateral triangular osteosynthesis and quantified fracture displacement based on relative changes between paired points across the fracture gap under seven loading scenarios, allowing a more direct evaluation of fracture-site stability and its Denis pattern–dependent variation.

The results demonstrated that the biomechanical behavior of TPO was not uniform across Denis fracture types. In Denis I fractures, fracture displacement in the plate group was markedly greater than that observed in the BTO construct, particularly under flexion and rotational loading conditions. As the fracture line progressively approached the sacral midline in Denis II and Denis III patterns, the magnitude of this displacement difference decreased substantially. In Denis III fractures, the mechanical discrepancy between the two fixation strategies became limited across most loading scenarios.

These findings support our initial hypothesis that the biomechanical performance of TPO is influenced by fracture morphology. Specifically, the reduction in displacement differences from Denis I to Denis III fractures indicates an increasing biomechanical compatibility between plate fixation and more medially located sacral fracture patterns.

Accordingly, the present findings should not be interpreted as establishing the superiority of one fixation method over another in absolute terms. Instead, they indicate that the biomechanical behavior of TPO should be interpreted in relation to fracture morphology, with BTO serving only as a high-stability reference construct. The present findings suggest that the biomechanical behavior of posterior trans-iliac plate fixation is strongly influenced by fracture morphology. As the fracture line progressively approached the sacral midline from Denis type I to Denis type III patterns, fracture displacement associated with TPO progressively decreased relative to the high-stability reference construct. This trend indicates increasing mechanical compatibility of TPO in more medially located sacral fracture patterns.

From a biomechanical perspective, the reduced difference between TPO and BTO observed in Denis type III fractures may be attributed to the anatomical characteristics of the central sacral canal involvement. In this fracture pattern, the continuity of the posterior sacral cortex is largely disrupted, and load transmission across the posterior pelvic ring becomes more dependent on global construct stiffness rather than local fracture bridging. As a result, the trans-iliac plate functions primarily as a tension-band structure spanning the bilateral ilia, partially compensating for the loss of intrinsic sacral stability. This mechanism may explain why the displacement discrepancy between the two fixation strategies decreased in Denis type III fractures compared with Denis type I and II patterns.

Taken together, these results emphasize that the key clinical question is not whether one fixation method is universally “stronger” or “weaker,” but how the mechanical characteristics of each construct align with fracture morphology, surgical invasiveness, and postoperative rehabilitation strategy. It should be noted, however, that the present simulations were performed using material properties corresponding to normal bone quality. Variations in bone mineral density, particularly in osteoporotic conditions, may alter construct performance and potentially influence the relative differences observed between fixation strategies. Future studies incorporating patient-specific bone quality parameters would therefore be valuable to further refine these biomechanical interpretations. By elucidating the Denis pattern–dependent mechanical compatibility of posterior trans-iliac plate fixation, the present study provides biomechanical reference data for further understanding the biomechanical characteristics of posterior trans-iliac plate osteosynthesis in unilateral vertically unstable sacral fractures with posterior pelvic ring instability.

## Limitations

This study has several limitations that should be acknowledged. First, the finite element models primarily focused on osseous structures, ligaments, and implant constructs, while active muscle forces were not incorporated. Although muscular loading may influence pelvic biomechanics under dynamic conditions, this simplification is commonly adopted in finite element studies aiming to evaluate immediate postoperative stability under controlled loading scenarios.

Second, all simulations were performed using a single lumbopelvic anatomical model. This approach was chosen to minimize variability related to individual differences in bone morphology and material properties; however, it does not fully represent the heterogeneity encountered in clinical populations, such as variations in bone quality, pelvic geometry, or fracture comminution.

Third, the fracture configurations investigated in this study were idealized representations of unilateral vertically unstable sacral fractures according to different Denis patterns. While these models allow systematic comparison of fixation behavior across fracture locations, they cannot encompass the full spectrum of complex injury patterns observed in clinical practice. In addition, the modeled TPO configuration represents a simplified posterior trans-iliac tension-band construct and does not encompass all clinically used reinforced fixation strategies. Similarly, BTO was included as a high-stability reference construct rather than as a clinically equivalent treatment alternative for all unilateral sacral fractures.

Furthermore, the present analysis focused on immediate postoperative mechanical stability under static loading conditions. The loading was applied in a quasi-static manner to represent physiological axial forces at neutral standing. Long-term biomechanical effects, including cyclic loading, implant fatigue, progressive fracture healing, and biological remodeling, were not addressed. Furthermore, time-dependent loading sequences (e.g., incremental load application at different simulation stages) were not explicitly evaluated. Future studies incorporating stepwise or cyclic loading protocols may provide further insight into stress evolution and construct durability over time. Therefore, the findings should be interpreted as reflecting early postoperative mechanical behavior rather than long-term clinical outcomes. Consequently, the present simulations should not be interpreted as direct evidence for postoperative weight-bearing recommendations or clinical superiority between fixation constructs.

Finally, although the lumbopelvic model used in this study was validated in our previous work against published cadaveric experimental data, it should be acknowledged that the reference experimental specimen was derived from an elderly donor, whereas the present model represents a healthy adult bone condition. Differences in bone quality, particularly in trabecular and subchondral bone properties, may influence absolute stress magnitudes. However, given that cortical bone plays a dominant role in load transmission within the pelvic ring, and that comparable cortical material parameters were adopted, the validation primarily supports the overall mechanical trends rather than exact numerical equivalence. Further investigations incorporating age-specific or osteoporotic bone properties would strengthen the translational relevance of these findings.

## Conclusions

In conclusion, the biomechanical behavior of TPO varied according to Denis fracture morphology. When interpreted relative to a high-stability BTO reference construct, the non-reinforced TPO construct used in this experiment showed larger fracture displacement in laterally located Denis I fractures, whereas the displacement discrepancy progressively decreased in Denis II and especially Denis III patterns. These findings provide biomechanical reference data for understanding fracture pattern–dependent fixation compatibility in unilateral vertically unstable sacral fractures. However, it remains unclear how much fixation strength is actually required to achieve sufficient construct stability for fracture healing and how postoperative management strategies, such as weight-bearing status, may influence this requirement.

## Data Availability

The datasets generated and/or analyzed during the current study are available from the corresponding author on reasonable request.
